# Sexual dimorphism in atherosclerotic plaques of aged *Ldlr*^−/−^ mice

**DOI:** 10.1186/s12979-024-00434-3

**Published:** 2024-05-02

**Authors:** Virginia Smit, Jill de Mol, Mireia N. A. Bernabé Kleijn, Marie A. C. Depuydt, Menno P. J. de Winther, Ilze Bot, Johan Kuiper, Amanda C. Foks

**Affiliations:** 1 LACDR, Leiden Academic Centre for Drug Research, Division of BioTherapeutics, Leiden University, Einsteinweg 55, 2333CC Leiden, The Netherlands; 2 Department of Medical Biochemistry, Amsterdam University Medical Centers – location AMC, University of Amsterdam, Experimental Vascular Biology, Amsterdam Cardiovascular Sciences, Meibergdreef 9, 1105 AZ Amsterdam, The Netherlands

**Keywords:** Cardiovascular disease, Atherosclerosis, Aging, Sex, Immunology, Single-cell transcriptomics

## Abstract

**Background:**

Atherosclerosis, the main underlying pathology of cardiovascular disease, is a chronic inflammatory disease characterized by lipid accumulation and immune cell responses in the vascular wall, resulting in plaque formation. It is well-known that atherosclerosis prevalence and manifestation vary by sex. However, sexual dimorphism in the immune landscape of atherosclerotic plaques has up to date not been studied at high-resolution. In this study, we investigated sex-specific differences in atherosclerosis development and the immunological landscape of aortas at single-cell level in aged *Ldlr*^−/−^ mice.

**Methods:**

We compared plaque morphology between aged male and female chow diet-fed *Ldlr*^−/−^ mice (22 months old) with histological analysis. Using single-cell RNA-sequencing and flow cytometry on CD45^+^ immune cells from aortas of aged *Ldlr*^−/−^ mice, we explored the immune landscape in the atherosclerotic environment in males and females.

**Results:**

We show that plaque volume is comparable in aged male and female mice, and that plaques in aged female mice contain more collagen and cholesterol crystals, but less necrotic core and macrophage content compared to males. We reveal increased immune cell infiltration in female aortas and found that expression of pro-atherogenic markers and inflammatory signaling pathways was enriched in plaque immune cells of female mice. Particularly, female aortas show enhanced activation of B cells (*Egr1*, *Cd83*, *Cd180*), including age-associated B cells, in addition to an increased M1/M2 macrophage ratio, where *Il1b*^+^ M1-like macrophages display a more pro-inflammatory phenotype (*Nlrp3*, *Cxcl2, Mmp9*) compared to males. In contrast, increased numbers of age-associated Gzmk^+^CD8^+^ T cells, dendritic cells, and *Trem2*^+^ macrophages were observed in male aortas.

**Conclusions:**

Altogether, our findings highlight that sex is a variable that contributes to immunological differences in the atherosclerotic plaque environment in mice and provide valuable insights for further preclinical studies into the impact of sex on the pathophysiology of atherosclerosis.

**Supplementary Information:**

The online version contains supplementary material available at 10.1186/s12979-024-00434-3.

## Introduction

Atherosclerosis, a chronic inflammatory disease characterized by lipid accumulation and immune cell infiltration in the arterial wall, is the main underlying pathology of cardiovascular disease (CVD). Although CVD is the leading cause of death in both women and men, accounting for 45% and 39% of all deaths respectively [1, 2], sex differences in atherosclerotic CVD prevalence and manifestation have been described. CVD develops about 10 years later in women than in men [3] but women have a poorer prognosis and are more likely to die following an acute cardiovascular event [4]. While acute cardiovascular events in women are mostly caused by stable atherosclerotic plaques that undergo erosion, in men, acute plaque rupture is often the culprit factor [5]. Moreover, women generally have smaller plaque area with decreased necrotic core volume compared to men [6, 7]. Incidence of thin-cap fibroatheroma and large calcification area varies by sex, but only when stratified by age, since men younger than 70 years of age showed a higher prevalence of thin-cap fibroatheroma and large calcification, while women older than 70 years showed a higher prevalence [8]. Notably, CVD risk in women is often missed due to the assumption that women are “protected” against CVD at younger age. Combined with the underrepresentation of women in scientific research, these factors contribute to a knowledge gap regarding the pathophysiology of atherosclerotic CVD in women [9].

Inflammation of the arterial wall is a key driver of atherosclerosis pathogenesis. Evidently, human and mouse studies that mapped the immune landscape of atherosclerotic plaques with single-cell technologies showed a heterogenous leukocyte pool within the plaque, including innate and adaptive immune cells [10–14]. Lymphoid cells, particularly T cells, were highly abundant in human atherosclerotic plaques and plaques of aged *Ldlr*^−/−^mice [15]. However, sexual dimorphism in the immune landscape of atherosclerotic plaques is seldomly studied. At a transcriptomic level, Hartman and colleagues reported significant sex-specific differences in sex-stratified gene regulatory networks from bulk RNA-sequencing derived from atherosclerotic aortic root tissue [16]. Genes that were more active in women were associated with mesenchymal and endothelial cells, while genes more active in men were associated with the immune system, particularly macrophages. Detailed profiling of plaque-residing immune cells is however lacking. Moreover, only few preclinical studies compared plaque immune cell numbers in the aortic root or arch between sexes, where either no differences were found between sexes or where increased infiltration of T cells in male chow diet-fed *ApoE*^−/−^mice was observed [17–20]. None of them have taken aging into account, one of the most dominant risk factors of CVD [21].

To bridge this knowledge gap, we investigated sex-specific differences in the atherosclerotic plaque of aged *Ldlr*^−/−^mice, a highly translational preclinical atherosclerosis model [15]. We compared plaque morphology between males and females with histological analysis. Using single-cell RNA-sequencing and flow cytometry on CD45^+^ immune cells from aortas of aged *Ldlr*^−/−^ mice, we explored the immune landscape in the atherosclerotic environment in males and females.

## Materials & Methods

### Animals

All animal experiments were approved by the Leiden University Animal Ethics Committee and were performed according to the guidelines of the European Parliament Directive 2010/63/EU. Male and female *Ldlr*^*−/−*^ mice on a C57Bl/6 J genetic background (3 months or 20 months old at the start of the experiment) were bred and aged in-house and kept under standard laboratory conditions. Young (3 months old) mice were randomized according to weight and basal serum cholesterol levels*,* and fed a regular chow diet (CD) or a Western diet (WD) containing 0.25% cholesterol and 15% cocoa butter (Special Diet Services, Witham, Essex, UK) for 10 weeks. Diet and water were provided ad libitum. At the end of experiment, mice were anaesthetized by a subcutaneous injection of a cocktail containing ketamine (100 mg/kg), atropine (0.5 mg/kg), and xylazine (10 mg/kg). Mice were bled by retro-orbital bleeding, and tissues were harvested after in situ perfusion with phosphate buffered saline (PBS). One mouse was excluded from the experiment due to presence of tumors.

### Histology

Hearts and aortas were embedded in O.C.T. compound (Sakura) and snap-frozen. To determine lesion size, cryosections (10 µm) of the aortic root were stained with Oil-Red-O and hematoxylin (Sigma-Aldrich). To quantify lesion volume, sections were collected from when aortic valves started to appear until a distance of 1.2 mm relative to the root was reached. The average of five sequential sections of the three-valve area of aortic roots, displaying the highest lesion content, was used to compare the vessel occlusion. Collagen content in the lesions was quantified using a Masson’s trichrome staining (Sigma-Aldrich). The necrotic core was defined as the acellular, debris-rich lesion area as percentage of total plaque area. Corresponding sections on separate slides were stained for monocyte/macrophage content with a MOMA-2 antibody (1:1000, AbD Serotec) followed by a biotinylated goat anti-rat IgG antibody (1:200, Vector). Secondary antibodies were detected using the Vectastain ABC kit (Vector) and visualized with ImmPACT NovaRED HRP substrate (Vector). We categorized cholesterol crystallization of atherosclerotic lesions in the aortic root on a scale of 0 (no cholesterol crystallization) to 3 (> 75% of the lesion area contains crystalline cholesterol). Presence of calcification was manually scored based on morphology. To quantify calcification area, sharp demarcated acellular dark pink to purple areas in the hematoxylin staining of three consecutive sections were divided by total plaque area [22]. Analysis and scoring were performed blinded. Mice with bicuspid aortic valves were excluded from histological analyses (*n* = 3). Pictures were taken with a Mikrocam II (Besser) linked to a Leica DM6000 Microscope. Stained sections were manually analyzed with ImageJ software.

### Aortic CD45^+^ cell isolation for single-cell RNA-sequencing

Atherosclerotic aortic arches, carefully detached from other surrounding organs, extensively flushed with PBS, and thoroughly cleaned from any residual perivascular adipose tissue, were isolated from aged chow diet-fed male *Ldlr*^*−/−*^mice (22 months old; *n* = 23) and enzymatically digested as previously described [15]. Single cell suspensions were stained with Fixable Viability DyeeFluor™ 780 (1:2000, eBioscience) and CD45-PE (1:500, clone 30-F11, Biolegend). After removing doublets, alive CD45^+^ cells were sorted (Supplementary Fig. 1) using a 100 µm nozzle in PBS supplemented with 0.04% BSA using a FACS Aria II SORP (BD Biosciences) and immediately processed for single-cell RNA-sequencing (scRNA-seq).

### Single-cell library preparation

Aortic CD45^+^ cell suspensions were loaded on a Chromium Single Cell instrument (10 × Genomics) to generate single cell gel bead emulsions (GEMs). ScRNA-seq libraries were prepared using the Single Cell 3^’^ Solution v2 Reagent Kit (10xGenomics). Sequencing was performed on an Illumina HiSeq2500 and the digital expression matrix was generated by de-multiplexing barcode processing and gene UMI (unique molecular index) counting using the Cell Ranger v6.0 pipeline (10 × Genomics).

### Single-cell data processing, integration, and analysis

The digital expression matrix of aortas isolated from chow diet-fed aged male *Ldlr*^−/−^ mice and of the female *Ldlr*^−/−^ mice, that was recently published [15], were analyzed using the R package Seurat (version 4). Cells were filtered by unique gene count per cell > 200 and < 6000 for aged male, and > 200 and < 7500 for aged female. In addition, a cutoff was set to a maximum of 6%, and 12% mitochondrial gene expression for aged male and aged female samples, respectively. Doublets were identified and removed using the DoubletDecon package. The remaining 5294 (aged male) and 4674 (aged female) cells were log-normalized, integrated using canonical correlation analysis and scaled subjected to principal component analysis (PCA). Based on the elbow plot, Jackstraw functions and separation of marker genes, 16 PCA components were included for cluster detection at a resolution of 0.245, which were subsequently visualized through Uniform Manifold Approximation and Projection (UMAP).

The Seurat function FindAllMarkers was used to find the differentially expressed genes (DEGs) per cluster, which were examined to define the cell clusters. For the high-resolution re-clustering, (*Cd79b*^+^) B-cell clusters, (*Cd3e*^+^) T-cell clusters and (*Cd68*^+^ and *Itgam*^+^) myeloid clusters were selected and extracted from the main clustering. Thresholds were set to Cd19 < 0.3, Cd79b < 0.3, Cd68 < 0.3 to exclude non-T-cells from the T-cell clustering, Cd3e < 0.3, Cd68 < 0.3 to exclude non-B-cells from the B-cell clustering, and Cd3e < 0.3, Cd19 < 0.3, Cd79b < 0.3 to exclude non-myeloid cells from the myeloid clustering. The variable genes of these selected clusters were then used as input for dimensionality reduction and re-clustering. PCA analysis on rescaled transcripts was performed with the following dimensions and resolutions: T cells (3155 cells), dimensions 9, resolution 0.6; B cells (2746 cells), dimensions 11, resolution 0.25; myeloid cells (1818 cells), dimensions 12, resolution 0.5. Tregs (Foxp3 > 0.3) and non-Tregs (Foxp3 < 0.3) were selected from cluster 4 CD4^+^T cells (Cd8a < 0.3, Cd8b1 < 0.3, Tcrg-C1 < 0.3, Cd4 > 0.4, Kit < 0.3). UMAP plots, dot plots, violin plots, volcano plots were generated in R. Enrichment scores of the SenMayo geneset were calculated using the AUCell package [23, 24]. Pathway analyses were performed using the Single Cell Pathway Analysis (SCPA) package [25].

### Flow cytometry

Immunostaining was performed as previously described on single cell suspensions derived from murine aortas to characterize immune cells [15]. To block Fc receptors, an unconjugated anti-CD16/32 antibody (clone 2.4G2, BD Bioscience) was used for mouse samples. Living cells were selected using Fixable Viability Dye-eFluor™ 780 (1:2000, eBioscience) and different cell populations were defined using anti-mouse fluorochrome-conjugated antibodies (Supplementary Table S1). Antibody staining of transcription factors and cytokines was performed using transcription factor fixation/permeabilization concentrate and diluent solutions and cytofix/permeabilization solutions, respectively (BD Biosciences). Flow cytometry analysis was performed on a Cytoflex S (Beckman Coulter) and the acquired data were analyzed using FlowJo software (version 10.7).

### Statistical analysis

Data are expressed as mean ± SEM. Outliers were identified and removed using Grubbs outlier tests (a = 0.05). Significance of data with more than 2 groups was tested using one-way ANOVA test followed by a Tukey multiple comparisons test. Statistical significance of data with 2 groups was tested using an unpaired two-tailed t-test or a nonparametric Mann–Whitney U test. Plotted comparisons are between males and females per age group. P-values of < 0.05 were considered significant. Statistical analysis was performed using GraphPad Prism 9.0.

## Results

### Atherosclerotic lesions of aged female mice are rich in collagen and cholesterol crystals

**Fig. 1:**
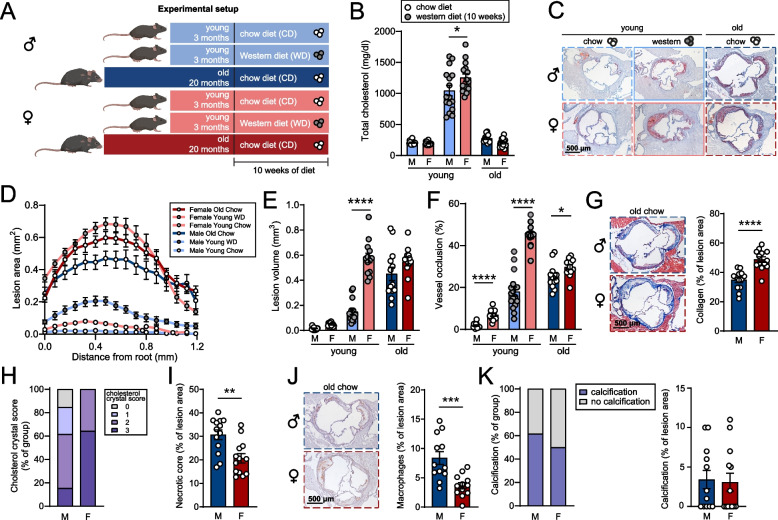
Plaque size and composition of aged male and female *Ldlr*^−/−^ mice. **A** Experimental setup: young male (light blue) and young female [15] (light red) *Ldlr*^−/−^ mice were randomized according to weight and basal serum cholesterol levels and fed a chow diet (white circles) or western diet (grey circles) for 10 weeks, and old male (dark blue) and old female [15] (dark red) *Ldlr*^−/−^ were fed a chow diet. **B** Total serum cholesterol levels at sacrifice were measured. **C** Cross sections of the aortic root were stained for lipid and collagen content. **D** Atherosclerotic lesion area over distance, **E** lesion volume, and **F** vessel occlusion were quantified. **G** Collagen content was quantified as percentage of lesion area. **H** Cholesterol crystallization in atherosclerotic lesions was categorized on a scale of 0 (no cholesterol crystallization) to 3. **I** Necrotic cores and **J** macrophage content (MOMA-2) were measured as percentage of lesion area. **K** Presence of calcification (purple) or no calcification (grey) was presented as percentage of the group and measured as percentage of lesion area. Data are from *n* = 12–16 mice per group. Statistical significance was tested by one-way ANOVA. Mean ± S.E.M. plotted. *P < 0.05, **P < 0.01, ***P < 0.001, ****P < 0.0001

The *Ldlr*^−/−^ mouse is a widely used experimental model to study atherosclerosis, but we and others have previously shown that severe hypercholesterolemia induced by a Western diet (WD) is needed to promote atherosclerosis in young (3 months) *Ldlr*^−/−^ mice (Fig. 1A-D) [15]. Notably, as shown in Fig. 1D-E, young female *Ldlr*^−/−^ mice are more prone to develop atherosclerosis compared to young male *Ldlr*^−/−^ mice upon WD feeding. However, this WD-accelerated induction of atherosclerosis in young mice diverges from the gradual buildup of atherosclerotic lesions and pathology that comes with aging as manifested in humans. We therefore investigated sex-related differences in atherosclerotic plaque development, composition, and the immune landscape in a more translational setting, using chow diet (CD)-fed aged (22 months) *Ldlr*^−/−^ mice (~ 200–250 mg/dl serum cholesterol, Fig. 1B) of both sexes. As opposed to the large discrepancy of lesion volume between sexes in the young WD-fed mice, lesion volume between aged male and female CD-fed mice did not statistically differ, although vessel occlusion was still slightly elevated in aged CD-fed females (Fig. 1E-F). Atherosclerotic lesions of aged female *Ldlr*^−/−^ mice were relatively enriched in collagen content and cholesterol crystals but showed less necrotic core and macrophage content compared to male mice (Fig. 1I-J). Calcification incidence and content, which significantly increases in aged atherosclerotic mice [15], were comparable between both sexes (Fig. 1K).

### Single-cell profiling reveals increased immune cell infiltration in the aorta of aged female mice

**Fig. 2:**
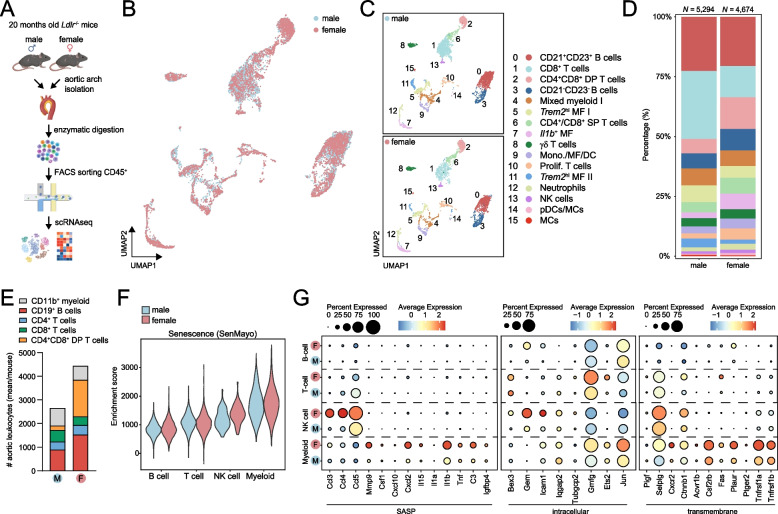
Immune cell landscape of aortas from aged male and female *Ldlr*^*−/−*^ mice**. A** Workflow of scRNA-seq on aortic CD45^+^ cells of chow diet-fed 22-months-old male (*n* = 23) and female (*n* = 12) *Ldlr*^−/−^ mice. UMAP visualization of clustered aortic leucocytes grouped by **B** sex or **C** immune cell clusters. **D** Stacked diagram showing the relative proportions of major immune cell subtypes within CD45^+^ cells of *Ldlr*^−/−^ aortas measured by scRNA-seq. **E** Stacked diagram showing the number of major immune cell types in the aorta of aged male and female *Ldlr*^−/−^, measured as mean per mouse with flow cytometry. **F** Violin plot showing the senescence (SenMayo gene set) enrichment score of major immune cell types per sex. **G** Average expression of SASP, intracellular and transmembrane genes from the SenMayo gene set in major immune cell types split by sex. DP, double positive; MF, macrophages; SP, single positive; DC, dendritic cell; NK, natural killer; pDC, plasmacytoid dendritic cell; MC, mast cell

Next, we sought out to explore sex differences in the immunological landscape of the aged atherosclerotic plaque and identify unique and conserved gene expression signatures of distinct plaque immune cell types between aged male and female mice. We performed single-cell RNA sequencing analysis on CD45^+^ cells obtained from the atherosclerotic aortic arch of aged female *Ldlr*^−/−^ mice [15], and integrated this with scRNA-seq data of aged male *Ldlr*^−/−^ mice (Fig. 2A). To identify distinct immune cell types in the atherosclerotic plaque of males and females, we performed dimensionality reduction and unsupervised cell clustering on a total of 9968 cells (male: 5294 cells, and female: 4674 cells). We observed overlapping alignment of the male and female immune cell clusters (Fig. 2B), indicating proper batch effect correction and consistency in cluster definition across sexes. Immune cell clusters were defined by canonical marker genes and visualized in a UMAP plot and proportional abundance barplot (Fig. 2C-D and Supplementary Figure S2A-C and Table S2). Proportionally, we observed increased abundance of CD8^+^ T cells in male aortas, while populations of CD4^+^CD8^+^ double positive (DP) T cells, CD21^−^CD23^−^ B cells and *Il1b*^+^ macrophages (MF) were increased in female aortas (Fig. 2D). We also measured sex-specific changes in major immune cell abundance with flow cytometry and found increased immune cell infiltration in the aortic arches of female mice (Fig. 2E). In agreement with the scRNA-seq data, male aortic arches showed increased numbers of CD8^+^ T cells and myeloid cells, whereas female aortic arches contained more CD19^+^ B cells and CD4^+^CD8^+^ DP T cells (Fig. 2E and Supplementary Figure S2D and S3).

To investigate potential sex-specific differences of senescence in the aortic leukocytes of these aged mice, we performed enrichment of the SenMayo senescence gene set [23]. Although B and T cells did not show any sex-specific difference, NK cells and to a lesser extent myeloid cells of female aortas displayed enrichment of senescence (Fig. 2F). In line with this, we observed increased expression of senescence-associated secretory phenotype (SASP) genes (e.g. chemokines, *Mmp9*, *Il1b, Tnf*), intracellular (e.g. *Gem*, *Icam1*, *Jun*) and transmembrane senescence-associated genes (e.g. *Cxcr2*, and STAT3 target genes *Tnfrsf1a/b*) in NK cells and myeloid cells of females (Fig. 2G). To gain further insight into possible sex differences within the subsets, we next performed reclustering of each major immune cell population (B cells, T cells and myeloid cells).

### Activated age-associated B cells are enriched in aortas of female mice

**Fig. 3:**
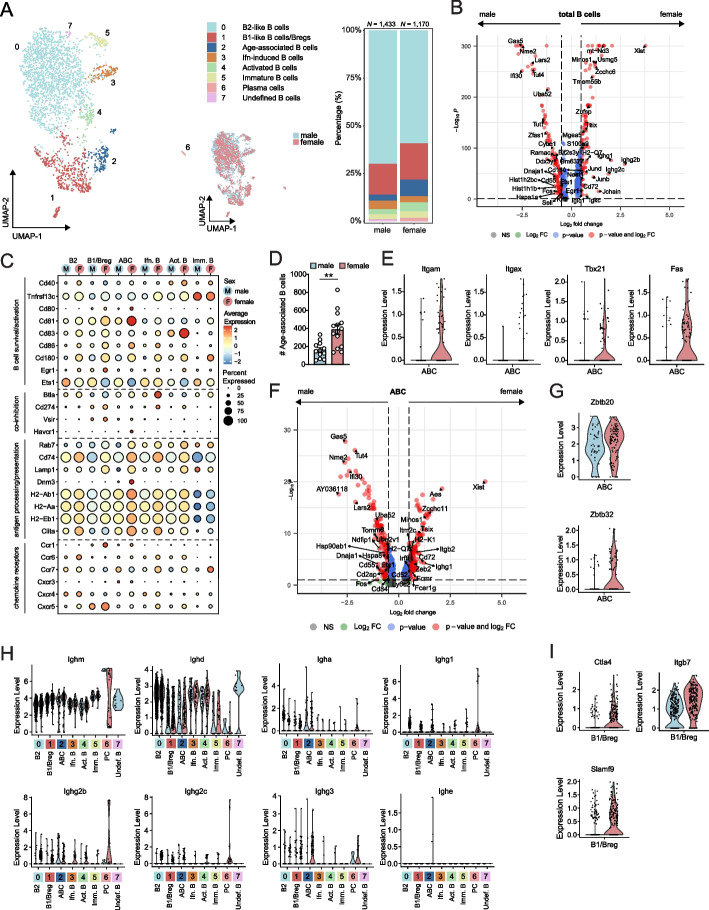
Sex-specific gene signatures of aortic B cells in aged *Ldlr*^−/−^ mice**. A** UMAP plots and stacked diagram of B cell clusters in the aorta of *Ldlr*^−/−^ mice. **B** Volcano plot displaying differentially expressed genes of the total B cell subclustering between aged male and female *Ldlr*^−/−^ mice. **C** Average expression of biological process-associated genes in B cell clusters split by sex. **D** Absolute number of age-associated B cells in the aortas of aged male and female *Ldlr*^−/−^ mice was measured with flow cytometry. **E** Sex-specific gene expression level of age-associated B cell-specific markers in cluster 2. **F** Volcano plot of cluster 2 displaying differentially expressed genes between male and female. Sex-specific expression level of **G** zinc-finger protein genes in cluster 2, **H** immunoglobulin isotype genes in all B cell clusters, and **I** genes differentially expressed in cluster 1. Flow cytometry data are from *n* = 12–14 mice per group. Statistical significance was tested by a t-test. Mean ± S.E.M. plotted. **P < 0.01

Proportionally, B2-like cells (cluster 0; *Ighd*, *Fcer2a*, *Cr2*) comprised the largest B cell cluster in aortas of both sexes (males 66% and females 54%) (Fig. 3A, Supplementary Figure S4 A and B, and Supplementary Table S3). We further detected B1-like cells and regulatory B cells (cluster 1; *Zbtb32*, *S100a6*, *Cd9*), age-associated B cells (ABCs, cluster 2; *Zbtb20*, *Tbx21*, *Fas*), Ifn-induced B cells (cluster 3; *Ifit2*, *Ifit3*, *Ifi213*), activated B cells enriched for Myc-target genes (cluster 4; *Nme2*, *Mif*), immature B cells (cluster 5; *Cd93*, *Cd24a*), plasma cells (cluster 6; *Sdc1*, *Jchain*, *Prdm1*) and undefined B cells (cluster 7).

Not surprisingly, DEG analysis showed upregulation of X-chromosomal genes (*Xist*, *Tsix*, *Gm6377*) in female B cells, while Y-chromosomal genes *Eif2s3y* and *Ddx3y* were upregulated in male B cells (Fig. 3B). Female B cells displayed upregulation of activation-related genes *Cd40*, *Cd80*, *Cd83*, *Cd86*, *Egr1*, *Cd180 *[26–30], while B cells in male aortas exhibited high expression of *Ets1*, a transcription factor that negatively controls B cell activation and concomitant antibody-secreting cell function [31], suggesting that B cells in males are less likely to contribute to humoral immunity (Fig. 3B and C). Additionally, expression of genes encoding co-inhibitory molecules was relatively higher in females compared to males, where *Havcr1* expression (Tim-1) was particularly high in the ABC cluster. Although hard to detect, both pro- and anti-inflammatory cytokine genes were mostly expressed at higher levels in female B cells (Supplementary Figure S4C). A similar pattern was seen in antigen-processing and presentation-related genes, particularly in ABCs. Strikingly, ABCs were more abundant in atherosclerotic aortic arches of aged female than in aged male *Ldlr*^−/−^ mice, which was confirmed with flow cytometry (386 ± 53 vs. 161 ± 26 cell count, *P* < 0.01; Figs. 3A and D). ABCs in females showed high expression levels of ABC-characteristic marker genes *Tbx21* (T-bet), *Fas*, and particularly *Itgax* (CD11c; Fig. 3E). Also, *Itgb2* (encoding CD18 that forms the functional CR4 complex with CD11c) and *Cd72* (encoding a transmembrane molecule that can regulate B cell activation) were upregulated in female ABCs (Fig. 3F) [32, 33]. *Cxcr3*, a chemokine receptor that is likely to be involved in the migration of B cells to the site of inflammation and differentiation into antibody-secreting plasma cells [34, 35], is almost exclusively expressed by the ABC cluster, but expression levels were comparable between sexes (Fig. 3C).

Certain zinc finger genes (*Zbtb20*, *Zbtb32*) in B cells are associated with plasma cell differentiation [36, 37], and expression levels of these genes were elevated in female ABCs (Fig. 3G). In line with this, plasma cells characterized by high expression of immunoglobulin-encoding genes and *Ly6c2 *[38], were more abundantly present in female aortas (Fig. 3A, H and Supplementary Figure S4D). Overall, immunoglobulin-encoding genes were more expressed in B cell clusters of females, of which ABCs showed high expression of *Ighg1* and *Ighg3* compared to other B cell clusters (excluding plasma cells; Fig. 3H). Notably, gene expression of *Ctla4* (co-inhibitory molecule), *Slamf9* (upregulated by inflammatory stimulus on B1 cells) and *Itgb7* (involved in homing of B cells) was increased in the female B1/Breg cluster [39–41], suggesting a more inflammatory and activated profile of the B1/Breg cluster in female mice (Fig. 3I).

### Granzyme-expressing effector CD8^+^ T cells are enriched in atherosclerotic aortas of aged males

**Fig. 4:**
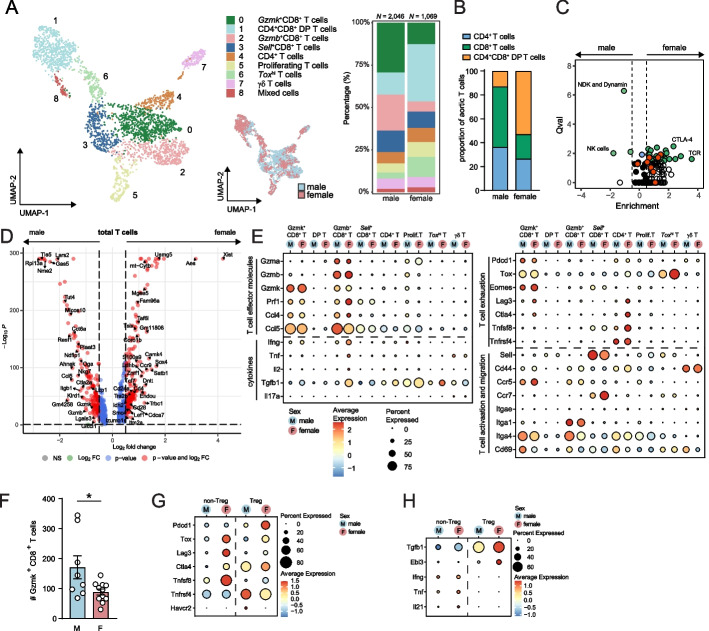
Transcriptomic comparison of T cells in aortas of aged male and female *Ldlr*^−/−^ mice. **A** UMAP plots and stacked diagram of T cell clusters in the aorta of *Ldlr*^−/−^ mice. **B** Stacked diagram showing the relative proportions of CD4^+^, CD8^+^ and CD4^+^CD8^+^ double-positive T cells within aged male and female *Ldlr*^−/−^ aortas, measured by flow cytometry. **C** Pathway enrichment of T cells in male and female mice. Green dots: significantly enriched pathways; red dots: interleukin-related pathways; white dots: insignificantly enriched pathways; black dots: insignificantly unenriched pathways. **D** Volcano plot displaying differentially expressed genes of the total T cell subclustering between aged male and female *Ldlr*^−/−^ mice. **E** Dot plot displaying the sex-specific expression of biological process-associated genes in T cell clusters. **F** Absolute number of *Gzmk*^*+*^CD8^+^ T cells in the aortas of aged male and female *Ldlr*^−/−^ mice was measured with flow cytometry. Average gene expression of **G** costimulatory and coinhibitory molecules and **H** cytokines in Tregs and non-Tregs from CD4^+^ T cells in cluster 4, split by sex

The aortic T cell pool of aged *Ldlr*^−/−^ mice contained 3 CD8^+^ T cell clusters, specifically *Gzmk*^+^CD8^+^ T cells (cluster 0; *Gzmk*, *Nkg7*, *Eomes*), *Gzmb*^+^CD8^+^ T cells (cluster 2; *Gzmb*, *Klrk1*, *Ly6c2*), and *Sell*^+^CD8^+^ T cells (cluster 3; *Sell*, *Klf2*, *Foxp1*; Fig. 4A and Supplementary Figure S5A and B and Table S4). CD8^+^ T cells comprised 63.3% of aortic T cells in males compared to 28.3% of aortic T cells in females (Fig. 4A). While CD4^+^CD8^+^ double positive (DP) T cells (cluster 1; *Rag1*, *Arpp21*, *Ccr9*) were the largest T cell cluster in the female aortic arches, the proportion of CD4^+^ T cells (cluster 4; *Tnfrsf4*, *Izumo1r*, *Icos*) did not differ between the sexes. These sex-specific frequencies of main T cell populations were also confirmed with flow cytometry (Fig. 4B). Additionally, we identified proliferating T cells (cluster 5; *Mki67*, *Pclaf*, *Nusap1*), *Tox*^*hi*^ T cells (cluster 6; *Tox*, *Itm2a*, *Nab2*), γδ T cells (cluster 7; *Tcrg-C1*, *Serpinb1a*, *Tmem176a*/*b*) and a cluster of mixed cells (cluster 8; *Malat1*, *Lck*).

Analysis on total T cells showed that, compared to females, the male T cell compartment was enriched in the natural killer pathway and cytotoxic/effector-related genes (*Nkg7*, *Ccl5*, *Klrd1*, *Gzmb*, *Gzmk*) (Fig. 4C-D). In contrast, CTLA-4, TCR and interleukin-related pathways (red dots in Fig. 4C) were more enriched in female T cells. Moreover, effector molecules (*Prf1*, *Ccl4*) and cytokines (*Ifng, Tnf, Il2, Tgfb1*) were expressed at higher levels in T cells from females than from males, particularly in the *Gzmb*^+^CD8^+^ T cells (Fig. 4E). *Gzmk*^+^CD8^+^ T cells were more abundant in males, as measured by scRNA-seq as well as with flow cytometry (Fig. 4A and F) and showed comparable gene expression of effector molecules and cytokines, but increased expression of some exhaustion markers (*Lag3*, *Ctla4*) in females (Fig. 4E). In addition, *Ccl5* and the gene encoding its receptor *Ccr5* were expressed on the majority of the *Gzmb*^+^ and *Gzmk*^+^CD8^+^ T cells. Interestingly, genes associated with T cell migration (*Itga1*, *Itga4*) and activation marker *Cd69* were expressed at higher levels in both granzyme-expressing CD8^+^ T cell clusters of males compared to females.

Cluster 4 mainly consisted of CD4^+^ T cells, including regulatory *Foxp3*^+^CD4^+^ T cells (Treg), but also contained some remainder *Kit*^+^ mast cells (Supplementary Figure S5B). In female atherosclerotic aortic arches, this cluster was enriched for *Lag3*, *Ctla4*, *Tnfsf8* (CD30L; Fig. 4E). Upon division of cells from cluster 4 into *Foxp3*^+^CD4^+^ Tregs and *Foxp3*^−^CD4^+^ non-Tregs, we found that Tregs in females showed higher expression of *Pdcd1* (PD-1), *Ctla4* and *Tnfrsf4* (OX40) but lower expression of *Havcr2* (TIM-3), while non-Treg CD4^+^ T cells in females specifically showed higher expression of *Tox*, *Lag3*, and *Tnfsf8* (Fig. 4G). This may indicate increased presence of the recently described CD30L^+^PD-1^+^CD44^+^CD4^+^ senescence-associated T cells [42] in aged aortas of female compared to male mice (Supplementary Figure S5C). Additionally, Tregs in females displayed higher expression of anti-inflammatory cytokine genes *Tgfb1* and *Ebi3* (IL-35), whereas non-Tregs in females showed elevated expression levels of *Tnf*, *Il18*, and *Il21* compared to non-Tregs in males (Fig. 4H).

### Female bias towards inflammatory M1-like macrophages in the aorta

**Fig. 5:**
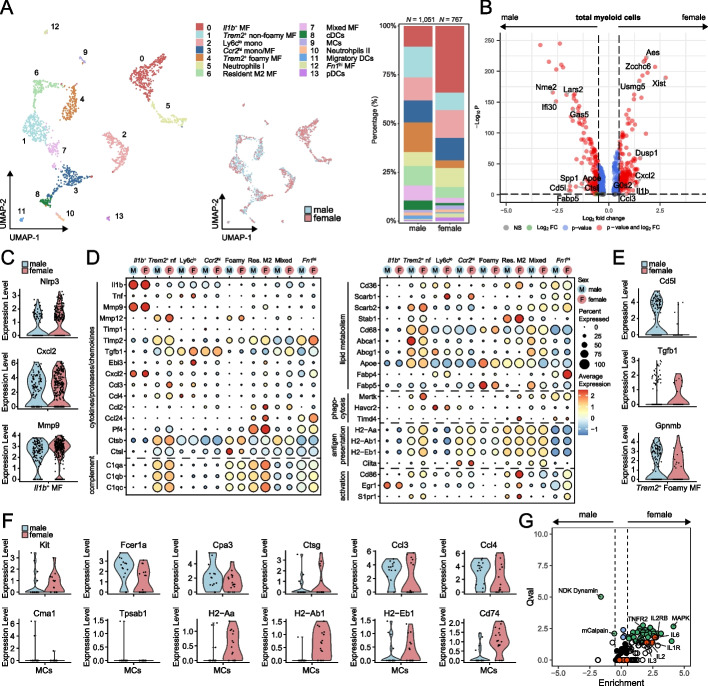
Plaques of aged male and female *Ldlr*^−/−^ mice differ in myeloid cell proportions**. A** UMAP plots and stacked diagram of myeloid cell clusters in the aorta of *Ldlr*^−/−^ mice. **B** Volcano plot displaying differentially expressed genes of the total myeloid cell cells between aged male and female *Ldlr*^−/−^ mice. **C** Sex-specific expression levels of *Il1b*^+^ M1-associated genes. **D** Dot plot displaying the sex-specific expression of biological process-associated genes in macrophage clusters. Sex-specific gene expression of **E** *Trem2*^+^ M2-related genes in cluster 4 and **F** mast cell associated-markers in cluster 9. **G** Pathway enrichment of myeloid cells in male and female mice. Green dots: significantly enriched pathways; red dots: interleukin-related pathways; white dots: insignificantly enriched pathways; black dots: insignificantly unenriched pathways

The aortic myeloid cell compartment contained M1- and M2-like macrophages, resident macrophages, dendritic cells, monocytes, neutrophils, and mast cells (Fig. 5A and Supplementary Fig. 6A and B). DEG analysis showed upregulation of *Cxcl2*, *Il1b* and *Ccl3* in aortic myeloid cells of *Ldlr*^−/−^ females, while *Fabp5*, *Apoe*, *Cd5l* and *Spp1* were upregulated in myeloid cells from *Ldlr*^−/−^ males (Fig. 5B). *Il1b*^+^ M1-like macrophages (*Il1b*, *Csf3r*, *Cxcr2*) were the most abundant myeloid population in females (~ 30% in females vs. ~ 10% in males). Moreover, expression of M1-like specific markers *Nlrp3*, *Cxcl2, Mmp9* was elevated in females, suggesting that these macrophages have an enhanced inflammatory phenotype in the atherosclerotic aorta of females compared to males (Fig. 5C). Males, on the other hand, show increased presence of *Trem2*^+^ myeloid cells including non-foamy M2-like macrophages (*Trem2*, *Mmp12*), foamy macrophages (*Fabp5*, *Cd5l*), resident M2 macrophages (*Lyve1*, *Mrc1*) and mixed *Trem2*^+^ macrophages (*Emp1*, *Lpl*; Fig. 5A and Supplementary Table S5). Foamy macrophages are characteristic of atherosclerotic plaques and are considered to be rather anti-inflammatory than pro-inflammatory [12]. *Cd36*, *Apoe, Fabp5*, and *Cd5l* expression was higher in male *Trem2*^+^ foamy macrophages, which mediate lipid-uptake and promote foam cell survival in lesions (Fig. 5D and E) [43, 44]. Additionally, male foamy macrophages showed increased expression of *Tgfb1* and *Gpnmb* (encoding a glycoprotein that is upregulated in foamy macrophages)[45], which have been described to regulate lesion development (Fig. 5E). Cluster 7 consists of a mix of foamy and non-foamy macrophages with differential expression of *Lpl* and *Spp1* between males and females (Supplementary Figure S6C).

Conventional dendritic cells (cDCs; *Xcr1*, *Ppt1*) and migratory dendritic cells (mDCs; *Ccr7, Ccl5, Ccl17, Cccl22*) were more abundant in males (Fig. 5A). Interestingly, male mDCs showed higher expression of chemokines *Ccl5* and *Ccl22* compared to females (Supplementary Figure S6D). Expression of MHCII-related genes *(H2-Aa*, *H2-Ab1*, *H2-Eb1*, *Ciita*) among the DC clusters was highest in the cDCs, but comparable between sexes (Supplementary Figure S6E). We identified cluster 5 and 10 as neutrophils (*Ly6g*, *Cd177*), of which cluster 10 seemed to be proliferating based on high expression of *Mki67* and histone-encoding genes (Supplementary Table S5). Although neutrophils in females showed elevated expression of pro-inflammatory gene *S100a8*, expression of other neutrophil markers were comparable (Supplementary Figure S6D). Mast cells (MCs) in cluster 9 showed comparable gene expression levels of MC-markers *Fcer1a* and *Cpa3*, while c-Kit (*Kit*) was more expressed in females (Fig. 5F). Although MC-specific protease genes encoding chymase (*Cma1*) and tryptase (*Tpsab1*) were barely detected, genes encoding secretory molecules *Ctsg*, *Ccl3*, *Ccl4* and antigen-presentation-associated molecules (*H2-Aa*/*Ab*/*Eb1*, *Cd74*) were increased in female MCs, suggesting a more pro-atherogenic signature of MCs in female compared to male aortas.

Lastly, pathway analysis showed enrichment of the phagocytosis-associated NDK Dynamin pathway (*Nme2*, *Dnm1*), and the migration-associated mCalpain pathway (*Cxcr3*, *Itgb1*, *Tln1*) in myeloid cells of males, while inflammatory signaling, such as interleukin-related (red dots: IL6, IL1R, IL2, IL2RB pathways), MAPK and TNFR pathways, was enriched in female myeloid cells (Fig. 5G).

## Discussion

Advances in single-cell technologies have enabled comprehensive profiling of immune cell populations in the atherosclerotic plaque. While sex is known to impact immune responses and atherosclerotic CVD prevalence and manifestation, sex differences in the immune landscape of the plaque are rarely studied. Our study reveals sexual dimorphism in plaque composition, immune cell proportions and gene signatures in aged *Ldlr*^−/−^ mice.

While plaques of young WD-fed male and female *Ldlr*^−/−^ mice show no difference in collagen and necrotic core area [46, 47], our data shows that plaques of aged female *Ldlr*^−/−^ mice were relatively more stable compared to plaques of aged male *Ldlr*^−/−^mice, due to increased collagen content, and less necrotic core area and macrophages. This corroborates with studies in humans, in which female CVD patients display similar signs of plaque stability compared to male [48]. We did however observe a higher influx of immune cells in female compared to male atherosclerotic aortas of aged *Ldlr*^−/−^mice and found that gene expression of pro-atherogenic markers and inflammatory signaling pathways were more enriched in female aortas. In line with these findings, women are known to elicit stronger innate and adaptive immune responses compared to men, contributing to their increased susceptibility for inflammatory and autoimmune diseases [49].

We report a striking increase of ABCs in aortas of atherosclerotic female mice, displaying enhanced expression of genes involved in B cell activation and antigen presentation, compared to ABCs in aortas of male atherosclerotic mice, which illustrates sex differences in B cell immunity that could contribute to atherosclerosis. We see enrichment of immunoglobulin genes in ABCs in addition to female-biased expression of *Tbx21* (T-bet) and *Cd72*, which are associated with autoantibody production [50–52]. Furthermore, expression of genes associated with plasma cell differentiation was elevated in female ABCs, suggesting that ABCs in females are more likely to become antibody-secreting cells in atherosclerosis. Accordingly, although only few plasma cells were found in the atherosclerotic aortas, their abundance was increased in females. High frequencies of ABCs in women have previously been linked to the susceptibility of autoimmune diseases, such as systemic lupus erythematosus, rheumatoid arthritis and multiple sclerosis [34, 35, 53–56]. Interestingly, both the TLR7 gene, crucial for ABC activation, and the gene for CD40L, which is involved in immunoglobulin class switching, are located on the X chromosome [57]. Since almost 15% of X-linked genes escape silencing, this may clarify the increased ABC frequency observed in females compared to males [58]. In addition, estrogen has been shown to stimulate the survival and activation of autoreactive B cells [59–61]. These findings contribute to the increasing body of evidence that atherosclerosis pathology involves autoimmune-like components [62–64], but where these age-associated B cells are precisely located in the atherosclerotic plaque environment remains to be investigated.

The presence of clonally expanded, activated T cells in the plaque of cardiovascular disease patients and mice also supports the concept of atherosclerosis as an inflammatory disease with autoimmune-like features [65, 66]. Depuydt et al. showed that clonally expanded CD8^+^ T cells in the plaque of male CVD patients had increased expression of granzymes (GZMB, GZMK and GZMA) compared to CD8^+^T cells in the blood [66]. Although we did not investigate clonality of T cells in this study, we show that the immune landscape in males is more CD8^+^ T cell-driven, illustrated by the large male-specific increase in *Gzmk*^+^CD8^+^ T cells and *Gzmb*^+^CD8^+^ T cells. Both CD8^+^ T cell populations express high levels of *Ccr5* and its ligand *Ccl5*, and in males show more expression of genes associated with activation and migration. Research has demonstrated that both antagonism and deficiency of the CCR5/CCL5-axis attenuate atherosclerosis in advanced stages by decreasing lesion size, promoting plaque stability, and reducing monocyte, macrophage, and T cell infiltration [67, 68]. The male-specific increase in *Ccr5*-expressing CD8^+^ T cells may contribute to the relatively increased macrophage content and reduced collagen that we observed in aged *Ldlr*^−/−^ male mice. Furthermore, we observed elevated gene expression of activation marker *Cd69* across multiple T cell types in males [69], corroborating with high expression of *Cd69* on clonally expanded T cells and a large proportion of CD69^+^cells among T cells in plaques of male CVD patients [66]. CD4^+^CD8^+^DP T cells accounted for the majority (~ 36%) of the T cells in females and have been previously found in murine and human plaques [13, 70]. Possibly, these cells escaped from the thymus into the periphery promoted by age-induced thymic involution [71]. However, in contrast to immature CD4^+^CD8^+^ thymocytes [72], CD4^+^CD8^+^ DP T cells in the plaque show high expression of the cytolytic factor GzmA and memory markers. In line with these findings, CD4^+^CD8^+^ DP T cells with cytotoxic or regulatory functions have been described in viral infections [73, 74], cancer [75, 76] and rheumatoid arthritis [77]. Although some studies show that sex hormones can influence thymic involution and the number of CD4^+^ CD8^+^ DP T cells [78–82], Aspinall et al. have shown a sex hormone-independent increase in CD4^+^ CD8^+^DP T cells in females [83].

We found an increase in CD11b^+^ myeloid cell numbers, including a larger proportion of conventional and migratory DCs, as well as *Trem2*^+^ non-foamy M2-like macrophages in aortas of aged males, while the female myeloid compartment largely contained pro-inflammatory *Il1b*^+^ M1-like macrophages. Elevated expression of foam cell survival genes in the male *Trem2*^+^ foamy macrophage cluster may explain the increase in foamy macrophage proportion and larger macrophage area observed in male lesions. In addition, increased expression of *Tgfb1* and *Gpnmb *in this cluster may contribute to regulating plaque development in the male mice [44]. We observed a higher M1/M2 macrophage ratio in atherosclerotic plaques of females than in males. In autoimmune diseases such as SLE and RA, females also show a bias towards M1 polarization, however the underlying mechanism is unclear [84]. Notably, mast cells displayed a more pro-atherogenic gene profile in female compared to male mice as illustrated by increased expression of proteases, chemokines and MHC class II molecules. This is in line with a previous study which showed that mast cells in females store and secrete more inflammatory mediators and are more likely to initiate an immune response [85].

Although limited conclusive information is available on how hormonal and chromosomal sex differences affect inflammation in atherosclerosis, a variety of studies highlighted the impact of estrogen on leukocyte migration. Estrogen inhibited IL-1-induced upregulation of ICAM-1 and VCAM-1 human endothelial cells [86], and reduced MCP-1 expression in rabbits [87]. These estrogen-related effects might decrease monocyte chemotaxis in atherosclerosis, thereby possibly leading to the lower macrophage content in females compared to males. Furthermore, men with androgen deficiencies have higher IL-1β concentrations than men with normal testosterone levels [88, 89], which might contribute to the lower proportion of inflammatory* Il1b*^+^ macrophages in males compared to females.

It should however be noted that female mice do not experience a dramatic reduction in estrogen levels that resembles human menopause, but have comparable estrogen levels during aging [90]. These endocrinologic differences between mice and men, in addition to dissimilarities in the aging environment between laboratory mice and humans, are limitations of using preclinical models [91–94].Apart from biological differences, it is important to keep in mind that our study faced several technical limitations. The limited number of aortic immune cells demands pooling of multiple samples to obtain enough events for single-cell RNA sequencing analysis, which restrained us from performing statistical analysis and may affect differential gene expression profiles. In addition, although we thoroughly cleaned and flushed the aorta, we cannot exclude contamination with a few circulating leukocytes. Nevertheless, our single-cell RNA sequencing analysis and validation at protein level using flow cytometry reveal an elaborate insight into immunological differences between aged atherosclerotic male and female mice, which should be taken into account in preclinical atherosclerosis research.

## Conclusion

Our data can be utilized as a valuable tool for future preclinical studies, including target validation in experimental mice for intervention studies, but also in refining study design and rationale for choosing the appropriate sex. Although we cannot directly extrapolate the observed sex differences in the murine atherosclerotic immune landscape to that of humans, we do see similarities between the aged *Ldlr*^−/−^ mouse model and human atherosclerosis pathology, illustrating the relevance of our data set.

Taken together, our study shows that sex is a variable that influences plaque characteristics and immune cell composition at single-cell resolution in aged *Ldlr*^−/−^ mice. These immunological sex differences may contribute to sex-based clinical differences in atherosclerotic CVD and highlight potential future areas of sex-specific immunomodulating therapies to combat atherosclerosis. To investigate this, further research into sex differences of the immune landscape of atherosclerotic plaques of cardiovascular disease patients is needed.

### Supplementary Information

**Additional File 1:** **Supplementary Figure S1**. Gating scheme of aortic CD45^+^ cells from male aged *Ldlr*^−/−^ mice before single-cell RNA sequencing. Gating strategy of alive aortic CD45^+^ cells for sorting from chow diet-fed aged male *Ldlr*^-/-^ mice. **Supplementary Figure S2.** Immune cell clustering and frequency in aortas of aged *Ldlr*^−/−^ mice. A) Heatmap of the top 50 differentially expressed genes (normalized single-cell gene expression shown) per cluster. B) Feature Dot Plot and C) Feature UMAP of the marker genes used for cluster annotation. D) Stacked diagram showing the relative proportions of major immune cell subtypes within aged male and female *Ldlr*^−/−^ aortas, measured by flow cytometry. **Supplementary Figure S3.** Biological distribution of immune cells in aged *Ldlr*^*−/−*^ mice. Flow cytometry analysis of CD11b^+^ myeloid, CD19^+^ B cells, CD4^+^ T cells, CD8^+^ T cells and double positive CD4^+^ CD8^+^ T cells in chow diet-fed aged male and female Ldlr^-/-^ mice. Data are from *n* = 12–14 mice per group. Statistical significance was tested by a t-test. Mean ± S.E.M. plotted. ***P< 0.001. **Supplementary Figure S4.** Sex-specific differences in aortic B cells of aged *Ldlr*^−/−^ mice. A) Average expression of cytokine and chemokine genes in B cell clusters split by sex. B) Feature Dot Plot and C) Feature UMAP of the marker genes used for cluster annotation. D) Sex-specific gene expression level of plasma cell-associated genes in B cell clusters. **Supplementary Figure S5.** Characterization of aortic T cells in aged *Ldlr*^−/−^ mice. A) Feauture Dot Plot of the marker genes used for cluster annotation. B) Average expression of canonical markers in T cell clusters projected on the UMAP plot. C) UMAP projection displaying sex-specific expression level of genes characteristic for senescence-associated CD4^+^ T cells. **Supplementary Figure S6.** Comparison of aortic myeloid cells between aged male and female *Ldlr*^−/−^ mice. A) Feature Dot Plot and B) Feature UMAP of the marker genes used for cluster annotation. Sex-specific expression of C) differentially expressed genes in cluster 7 and D) chemotaxis genes specific for migratory dendritic cells. E) Average expression of MHCII-related genes in dendritic cell clusters split by sex. F) Average expression of neutrophil markers in myeloid cell clusters split by sex.

## Data Availability

In silico data analysis was performed using custom R scripts (R version 4.1.2) designed especially for this research and/or based on the recommended pipelines from the pre-existing packages listed in the individual segments above. Single-cell RNA sequencing data are available upon personal request from the corresponding author (a.c.foks@lacdr.leidenuniv.nl).
