# Supplementary material for: Sexual dimorphism in atherosclerotic plaques of aged Ldlr−/− mice
Source: Immun Ageing. 2024 May 2;21:27. doi: 10.1186/s12979-024-00434-3 (PMC11064395; doi:10.1186/s12979-024-00434-3)
Supplement: Supplementary file 1 — Additional File 1: Supplementary Figure S1. Gating scheme of aortic CD45+ cells from male aged Ldlr−/− mice before single-cell RNA sequencing. Gating strategy of alive aortic CD45+ cells for sorting from chow diet-fed aged male Ldlr-/- mice. Supplementary Figure S2. Immune cell clustering and frequency in aortas of aged Ldlr−/− mice. A) Heatmap of the top 50 differentially expressed genes (normalized single-cell gene expression shown) per cluster. B) Feature Dot Plot and C) Feature UMAP of the marker genes used for cluster annotation. D) Stacked diagram showing the relative proportions of major immune cell subtypes within aged male and female Ldlr−/− aortas, measured by flow cytometry. Supplementary Figure S3. Biological distribution of immune cells in aged Ldlr−/− mice. Flow cytometry analysis of CD11b+ myeloid, CD19+ B cells, CD4+ T cells, CD8+ T cells and double positive CD4+ CD8+ T cells in chow diet-fed aged male and female Ldlr-/- mice. Data are from n = 12–14 mice per group. Statistical significance was tested by a t-test. Mean ± S.E.M. plotted. ***P< 0.001. Supplementary Figure S4. Sex-specific differences in aortic B cells of aged Ldlr−/− mice. A) Average expression of cytokine and chemokine genes in B cell clusters split by sex. B) Feature Dot Plot and C) Feature UMAP of the marker genes used for cluster annotation. D) Sex-specific gene expression level of plasma cell-associated genes in B cell clusters. Supplementary Figure S5. Characterization of aortic T cells in aged Ldlr−/− mice. A) Feauture Dot Plot of the marker genes used for cluster annotation. B) Average expression of canonical markers in T cell clusters projected on the UMAP plot. C) UMAP projection displaying sex-specific expression level of genes characteristic for senescence-associated CD4+ T cells. Supplementary Figure S6. Comparison of aortic myeloid cells between aged male and female Ldlr−/− mice. A) Feature Dot Plot and B) Feature UMAP of the marker genes used for cluster annotation. Sex- [file 12979_2024_434_MOESM1_ESM.pdf]

Sexual dimorphism in atherosclerotic plaques of aged *Ldlr*$^{-/-}$ mice

Virginia Smit[1], Jill de Mol[1], Mireia N. A. Bernabé Kleijn[1], Marie A.C. Depuydt[1], Menno P.J. de Winther[2], Ilze Bot[1], Johan Kuiper[1], and Amanda C. Foks[1]

1. Leiden Academic Centre for Drug Research, Division of BioTherapeutics, Leiden University, Einsteinweg 55, 2333 CC Leiden, The Netherlands.
2. Amsterdam University Medical Centers – location AMC, University of Amsterdam, Experimental Vascular Biology, Department of Medical Biochemistry, Amsterdam Cardiovascular Sciences, Meibergdreef 9, 1105 AZ Amsterdam, The Netherlands.

Correspondence to:

Amanda C. Foks, PhD

Division of BioTherapeutics

Leiden University, LACDR

Einsteinweg 55, 2333CC Leiden

The Netherlands

Tel; +31 (0) 71-5276213

a.c.foks@lacdr.leidenuniv.nl

Supplementary Tables

Supplementary Table S1. Major Resources Table

Animals (in vivo studies)

Species	Vendor or Source	Background Strain	Sex	Persistent ID / URL
Mouse, *Ldlr*$^{-/-}$	Jackson	C57BL/6J	Male	www.jax.org
Mouse, *Ldlr*$^{-/-}$	Jackson	C57BL/6J	Female	www.jax.org

Antibodies

Target antigen	Vendor or Source	Catalog #	Working concentration	Persistent ID / URL
**Mouse**				
MOMA-2	Bio-Rad (formerly AbD Serotec)	MCA519G	1:1000	www.bio-rad-antibodies.com
α-Rat	Vector	BA-4001	1:200	www.vectorlabs.com
CD4 – V500	BD Biosciences	560782	1:1000	www.bdbiosciences.com
CD8a – AF700	Biolegend	100730	1:500	www.biolegend.com
CD19 – BV605	Biolegend	115540	1:500	www.biolegend.com
CD11b – PE	eBioscience	12-0112-82	1:1000	www.thermofisher.com/ebioscience
CD11c – FITC	Biolegend	117306	1:800	www.biolegend.com
CD8a – PE-Texas Red	Invitrogen	MCD0817	1:1000	www.thermofisher.com/invitrogen
CD45 – AF700	Biolegend	103128	1:1000	www.biolegend.com
CD3 – eFluor450	eBioscience	48-0032-82	1:200	www.thermofisher.com/ebioscience
CD11b – BV605	Biolegend	101257	1:500	www.biolegend.com
PD-1 – PerCP.Cy5.5	Biolegend	109120	1:400	www.biolegend.com
Ly6C – APC	eBioscience	17-5932-82	1:500	www.thermofisher.com/ebioscience
CD45 – AF700	Biolegend	103128	1:1000	www.biolegend.com

Tox – PE	Miltenyi Biotec	130-120-716	1:400	www.miltenyibiotec.com
CD45 – PE	Biolegend	103106	1:500	www.biolegend.com
CD16/32 (Fc Block)	BD Biosciences	553142	1:250	www.bdbiosciences.com
Fixable viability dye – eFluor 450	eBioscience	65-0865-18	1:2000	www.thermofisher.com/ebioscience

Other

Description	Source / Repository	Persistent ID / URL
Trichrome Stain (Masson) Kit	Sigma Aldrich	www.sigmaaldrich.com
Oil Red O	Sigma Aldrich	www.sigmaaldrich.com
Hematoxylin Solution, Mayer's	Sigma Aldrich	www.sigmaaldrich.com
RPMI 1640	Gibco	www.thermofisher.com
Vectastain ABC kit (PK-4000)	Vector	www.vectorlabs.com
ImmPact NovaRed kit	Vector	www.vectorlabs.com
Collagenase I	Sigma Aldrich	www.sigmaaldrich.com
Collagenase XI	Sigma Aldrich	www.sigmaaldrich.com
Hyaluronidase	Sigma Aldrich	www.sigmaaldrich.com
DNAse I	Sigma Aldrich	www.sigmaaldrich.com
Fetal Bovine Serum	Greiner Bio-One	www.gbo.com

Supplementary Table S2. Top 25 differentially expressed genes Main clustering

**Main.00**	**Main.01**	**Main.02**	**Main.03**	**Main.04**	**Main.05**	**Main.06**	**Main.07**
Ighd	Ccl5	Rag1	Ighm	Fn1	C1qa	Lef1	Il1b
Ebf1	Nkg7	Arpp21	Mzb1	Ifitm3	C1qb	Itm2a	Retnlg
Cd79a	Ms4a4b	Dntt	Iglc2	Ccr2	C1qc	Tcf7	Csf3r
Cd79b	AW112010	Satb1	Ms4a1	Psap	Apoe	Tmsb10	S100a8
Mef2c	Ctla2a	Ccr9	Iglc1	Tgfbi	Pf4	Trac	Ifitm1
Ms4a1	Ctsw	Cd8b1	C130026I21Rik	Ms4a6c	Ms4a7	Satb1	Slpi
Fcmr	Hcst	Endou	AC168977.1	Ccl9	Selenop	Cd27	Msrb1
Bank1	Ms4a6b	Ldhb	Pkig	Lgals3	Mrc1	Cd28	Cxcr2
Iglc3	H2-Q7	Rmnd5a	Iglc3	Plbd1	Mafb	Cd3d	G0s2
Iglc2	Klrd1	Tcf7	Cd79a	Naaa	Ctsb	Cd5	Mmp9
Ly6d	Il2rb	Trbc2	Ly6a	Mpeg1	Trem2	Prkcq	Hdc
Fcer2a	Xcl1	Sox4	H2-DMb2	Gm2a	Trf	Rgs10	Stfa2l1
H2-DMb2	Gzmk	Cd8a	Cd79b	Alox5ap	Dab2	Tox	Hp
Cd74	Gzmb	Themis	Napsa	Ifitm2	Cd63	Trbc2	Ccl6
H2-Ob	Cxcr6	Ssbp2	Tcf4	Ctss	Lgmn	Txk	Mxd1
Iglc1	Gimap4	Lck	Igkc	H2-DMb1	Cxcl16	Cd3g	Ifitm2
Vpreb3	Cd3g	Myb	Plac8	Cst3	Csf1r	Bcl11b	Tnfaip2
Cd55	Klrk1	Cd4	Fcmr	Lyz2	Fcrls	Cd6	Grina
Pax5	Bcl2	Arl5c	Ly6d	S100a4	Cd14	Ccr9	Slc7a11
Gm31243	Gimap3	Aqp11	Zbtb20	Vim	Timp2	Cd2	Ccr1
Ralgps2	Gm2682	Mier1	D10Wsu102e	Fabp5	Lpl	Chd3	Gsr
H2-Aa	Gimap7	Bcl11b	Dnajc7	Lyz1	Adgre1	Sox4	Dusp1
Tnfrsf13c	Trac	Tcf12	Zcwpw1	Ifi30	Pltp	Ets1	S100a9
H2-Oa	Itgb1	Trbc1	Tppp3	Plac8	Hexb	Trbc1	Cxcl2

H2-Eb1	Ly6c2	Gm4258	Crip1	Retnla	Mmp12	Ikzf2	S100a11

**Main.08**	**Main.09**	**Main.10**	**Main.11**	**Main.12**	**Main.13**	**Main.14**	**Main.15**
Trdc	Ear2	Stmn1	Cd5l	Camp	Ncr1	Siglech	Fscn1
Tmem176a	Ace	Top2a	Cd63	Ngp	Klrb1c	Cox6a2	Cacnb3
Tmem176b	Clec4a3	Mki67	Gpnmb	Ltf	Klra4	Klk1	Ccl22
Tcrg-C1	Eno3	Hist1h2ae	C1qb	Lcn2	Klra8	Gm21762	Socs2
Cd163l1	Treml4	Hist1h1b	C1qc	Wfdc21	Klrb1b	Fcer1a	Zmynd15
Cxcr6	Cd300e	Pclaf	C1qa	Chil3	Klrb1a	Gata2	Tbc1d4
Il18r1	Csf1r	Ube2c	Fabp5	Mmp8	Klra9	Cpa3	Anxa3
Actn2	Grk3	Hist1h3c	Trem2	Ifitm6	Klre1	Smim5	Rogdi
Trdv4	Clec4a1	Birc5	Mmp12	Cd177	Klrc2	Atp1b1	Cxcl16
Podnl1	Adgre4	Rrm2	Apoe	Mmp9	Klra7	Cd7	Serpinb6b
Kcnk1	Dusp16	Cenpf	Ftl1	Ly6g	Klrk1	Cyp11a1	Ccr7
Ltb4r1	Cx3cr1	Nusap1	Ms4a7	Adpgk	Prf1	Plac8	Relb
Ly6g5b	Lst1	Hist1h2ap	Fth1	Mcemp1	Gzma	Tyrobp	Basp1
Il7r	Pla2g7	H2afx	Lgals3	Cebpe	Serpinb9	Csf1	Fabp5
Tmem64	Clec4e	Dut	Wfdc17	Mgst1	Gzmb	Rnase6	Gadd45b
Maf	Ms4a6c	Hmgn2	Spp1	Hp	Klrd1	Ctsl	Tmem176a
Ckb	Tnfrsf1b	Anp32e	Ctsb	Retnlg	Il2rb	Tcf4	Etv3
Ramp1	Gngt2	Ptma	Ctsl	Anxa1	Serpinb6b	Ifitm1	Syngr2
S100a4	Cebpb	Hmgb1	Cd68	Dstn	Ctsw	Bst2	Traf1
Serpinb1a	Nr4a1	H2afz	Serpinb6a	Pglyrp1	Nkg7	Hdc	Samsn1
Lmo4	Fcer1g	Hmgb2	Ctsd	S100a8	Cd7	Ccl4	Tmem123
Blk	Cybb	Tuba1b	Selenop	S100a9	AW112010	Irf8	Marcks
Emb	Sat1	H2afv	Cstb	Prdx5	Xcl1	Ccl3	Map4k4
Ifngr1	Plek	Ube2s	S100a1	Cybb	Ccl5	Ccl9	Cst3
Cd3g	Prdx1	Tubb5	Ctss	Lyz2	Ccl4	Ccl6	Ifi30

Supplementary Table S3. Top 25 differentially expressed genes B cell clustering

**B.00**	**B.01**	**B.02**	**B.03**	**B.04**	**B.05**	**B.06**	**B.07**
Ighd	S100a6	Nt5e	Ifit3	Srm	Akap12	Derl3	Fchsd2
Fcer2a	Ahnak	AC168977.1	Ifi213	Nop58	Atp1b1	Fam46c	Gm20559
Mef2c	Zbtb32	AC133103.1	Usp18	Ncl	Fam129c	Jchain	Zfp329
Satb1	Ahnak2	Itm2c	Ifit2	C1qbp	Vpreb3	Iglv1	Chd2
Neurl3	Lmna	C130026I21Rik	Irf7	Nme1	Myb	Slpi	C1qa
Ralgps2	Anxa2	Itgb1	Slfn5	Hsp90ab1	Pafah1b3	Mt1	BE692007
Stk17b	Rassf4	Adgre1	Ifi206	Eif4a1	Cd79b	Ly6c2	Slc38a2
Fchsd2	Vim	Zbtb20	Ifi203	Ddx21	Sox4	Creld2	Gm31243
Cd55	Tppp3	Bhlhe41	Zbp1	Mif	Ly6d	Tmem176b	Cd69
Cr2	Myadm	Fgl2	Ifi27l2a	Nhp2	Cd24a	Prdx4	Macf1
Sell	Itgb1	Ighg3	Ifi214	Gnl3	Spib	Edem1	Plk2
H2-Ob	Pdlim1	Zeb2	Trim30a	Ranbp1	Cecr2	Xbp1	Zfp869
Pxdc1	Lgals1	Txn1	Stat1	Npm1	Iglc1	Sdf2l1	Atxn2
Pxk	S100a4	H2-Q7	Ms4a4c	Ppp1r14b	Hck	Txndc5	Zcchc7
Zfp318	Crip1	Tcf4	Shisa5	Hspe1	Siglecg	Pdia4	Pan2
Pold4	Tagln2	Fcgr2b	Ifi208	Dkc1	Chchd10	Igkc	Mycbp2
Gm31243	Atf3	Lgals1	Xaf1	Set	Arl5c	Ighg2c	Kidins220
Smad7	Plac8	Pld4	Ifi209	Eif5a	Cnp	Ssr4	2810013P06Rik
Icosl	Ccnd2	Apoe	Ifi47	Fbl	Dnajc7	Sec11c	Slc12a6
Gm8369	S100a11	Ptpn1	Rnf213	Hspd1	Tifa	Hsp90b1	Usp15
Bmyc	S100a10	Igha	Parp14	Pa2g4	Tcf3	Manf	Txndc16
Dmxl1	Zcwpw1	Tnfaip8	Phf11b	Psme2	Bcl7a	Mzb1	Akap9

Lmo2	Cd2	Fcer1g	Samhd1	Hspa5	Rgs2	Slc3a2	Tut7
H2-Eb2	Zbtb20	Cd72	Bst2	Il4i1	Marcks	Ighg1	Arid1a
Tecpr1	D10Wsu1<br>02e	Xist	Tor3a	Ccnd2	Ifi30	Ighg2b	Cxcr4

Supplementary Table S4. Top 25 differentially expressed genes T cell clustering

T.00	T.01	T.02	T.03	T.04	T.05	T.06	T.07	T.08
Gzmk	Rag1	Gzmb	Sell	Tnfrsf4	Pclaf	Itm2a	Tmem176a	Malat1
Nkg7	Arpp21	Klrk1	Rps20	Gpm6b	Hist1h3c	Rgs10	Tmem176b	Lck
Ctla2a	Dntt	Itga1	Rps19	Izumo1r	Rrm2	Lef1	Tcrg-C1	Prrc2c
Ccl5	Endou	Klrd1	Ccr7	Maf	Birc5	Satb1	Serpinb1a	Ptprc
Ms4a4b	Rmnd5a	Ccl5	Rpl23	Ifi27l2a	Nusap1	Sox4	Blk	Mbnl1
Eomes	Ldhb	Nkg7	Rps16	Ly6a	Cenpf	Tox	Actn2	mt-Nd4
Hcst	Ccr9	Ly6c2	Rplp1	Capg	Mki67	Gsn	Pxdc1	mt-Cytb
AW112010	Aqp11	AW112010	Rpl13	Icos	Hist1h2ab	Ccr9	Kcnk1	Ccr9
Rpl13a	Myb	Fgl2	Rps28	Tnfsf8	Ube2c	Cnn3	Ltb4r1	Mier1
Itga4	Satb1	Ctsw	Rps24	Tnfrsf18	Lig1	Tmsb10	Trdv4	Arpp21
H2-K1	Ssbp2	Lgals3	Rps7	Pou2f2	Top2a	Basp1	Cd163l1	Ets1
Rgs1	Arl5c	Zeb2	Rps18	Shisa5	Stmn1	Cd28	Ckb	Srrm2
H2-Q7	Sox4	Ahnak	Dapl1	Hif1a	Dut	Cd2	Podnl1	Tnrc6b
Ccr5	Cd4	Itgb1	Ms4a4c	Eea1	Hist1h2ae	Id3	Igf1r	Cd164
Ms4a6b	Dgkeos	Cx3cr1	Rpl36a	Nrp1	Tuba1b	Ikzf1	Capg	Son
Pdcd1	Mier1	Klre1	Rpl12	Cd82	Hmgn2	Cd5	Maf	Akap13
Tox	Ets2	Gzma	Rpl39	Ctla4	Ptma	Ikzf2	Trdc	Clk1
Gimap7	Tcf7	Klrc1	Rps12	Tbc1d4	Hist1h1b	Cd27	Il18r1	Thrap3
Bcl2	Themis	Cd226	Rps5	S100a11	H2afx	Hivep3	Selenop	Ppp2ca
Gm8369	Gtf2h4	Id2	Eef1b2	Ltb	Ran	Xist	Ccr2	Kpna4
Ccl4	Xrcc6	S100a6	S1pr1	Srgn	Hist1h2ap	Etnk1	Lmo4	Selenos

Zfp36l2	Tcf12	Lgals1	Klf2	Odc1	Hmgb2	Cytip	S100a4	Itgb1
Runx3	Cyb5a	Cd48	Igfbp4	Junb	H2afz	Sept7	Il7r	Lyz2
BE692007	Ly6d	Ccl4	Ly6c2	Ikzf2	Tubb5	Egr1	Cxcr6	Tnrc6c
Cst7	Gm4258	Xcl1	Gzmm	Samhd1	Hist1h4d	Trbc1	S100a6	Wbp11

Supplementary Table S5. Top 25 differentially expressed genes myeloid cell clustering

**My.00**	**My.01**	**My.02**	**My.03**	**My.04**	**My.05**	**My.06**
Cxcr2	Nes	Cd300e	Ms4a4c	Cd5l	Itgb2l	Cbr2
Csf3r	Cd72	Treml4	Ccr2	Ftl1	Ly6g	Mgl2
Hdc	Cadm1	Ace	Vcan	Fth1	Cd177	Cd163
Il1b	Ms4a7	Eno3	S100a4	Atp6v0d2	Ngp	Mrc1
H2-Q10	Myo1e	Adgre4	Fn1	Fabp5	Ltf	Lyve1
Mmp9	Zmynd15	Ear2	Plac8	Lgals3	Wfdc21	Igfbp4
Mxd1	Cxcl16	Spn	Ms4a6c	Gpnmb	Adpgk	Gas6
Msrb1	C3ar1	Dusp16	Tmsb10	Apoe	Chil1	Pf4
Junb	Trem2	Pou2f2	Itgb7	Syngr1	Lcn2	C4b
Slpi	Igf1	Grk3	Ms4a6b	Cd63	Camp	Folr2
Dusp1	Slamf9	Gngt2	Ifitm3	Mmp12	Cebpe	Stab1
Ccr1	Mafb	Nr4a1	Napsa	Trem2	Ckap4	Maf
Srgn	C1qa	Itgal	Slfn5	Serpinb6a	Ly6c2	F13a1
S100a11	C1qc	Itga4	Ly6c2	Lgals1	Mmp8	Fcrls
Fgl2	Hexb	Myo1g	H2-DMa	Cstb	Serpinb1a	Cd209f
Sell	Gatm	Cybb	H2afy	Ctsd	Ifitm6	Dab2
Ifitm1	C1qb	Clec4a3	Ifi209	Ctsl	S100a8	Ccl8
Grina	Mmp12	Clec4a1	Ahnak	Wfdc17	S100a9	Ccl12
Slc7a11	Clic4	Stap1	S100a10	Igf1	Pglyrp1	Tmem176b
Il1r2	Anxa5	Tnfrsf1b	Crip1	Spp1	Syne1	Cfh
Lrg1	Lmna	Bcl2a1d	Vim	Il18bp	Mmp9	Selenop
Stfa2l1	Lpl	Ms4a6c	Ifi27l2a	Pld3	Anxa1	Fcgrt
Ptgs2	Rgs1	Apoc2	Lyz2	AY036118	Chil3	Ctsc
G0s2	Ctsb	Txnrd1	Ifi30	Ftl1-ps1	Retnlg	Trf
Cxcl2	Apoe	Prdx1	Thbs1	S100a1	Hmgn2	Ccl7

**My.07**	**My.08**	**My.09**	**My.10**	**My.11**	**My.12**	**My.13**
Emp1	Xcr1	Gata2	Birc5	Cacnb3	Tgfb2	Gm21762
S100a10	Sept3	Cpa3	Hist1h3c	Fscn1	Ltbp1	Klk1
Fn1	Itgae	Csrp3	Fcnb	Ccr7	Prg4	Atp1b1
Psap	Ifi205	Fcer1a	Pclaf	Ccl22	Ptgis	Cox6a2
Capg	Olfm1	Ms4a2	Ube2c	Socs2	Alox15	Siglech
F10	Wdfy4	Cyp11a1	Mki67	Serpinb6b	Selp	Upb1
Apoc2	Naaa	Cd200r3	Top2a	Apol7c	Ednrb	Spib
Cstb	Naga	Sytl3	Elane	Serpinb9	Saa3	Ccr9
Lgals3	Irf8	Mcpt8	Cebpe	Tbc1d4	Fcna	Iglc3
Gpnmb	Plbd1	Il6	Hist1h1b	Clu	Serpinb2	Cd7
Plin2	Psmb9	Nedd4	Hist1h2ae	Il4i1	Icam2	Smim5
Ctss	Cst3	Cd7	Stmn1	Relb	Flnb	Bcl11a
Ifi30	Gng10	Ctsg	Serpinb1a	Zmynd15	C4b	Rpgrip1
Lgals1	H2-DMb1	Il18r1	Ltf	Traf1	Ltc4s	Ly6d
Ahnak	Ckb	Cd69	Camp	Birc2	Ecm1	Runx2
Atp6v0c	H2-DMa	Csf1	Hist1h2ap	Ccl5	Fn1	Fyn
Lgmn	Ppt1	Ifitm1	Ngp	Tmem123	Fabp4	Rnase6
Lpl	Psmb8	Ccl4	Chil3	Anxa3	Itga6	Ly6a
Ccl9	Eef1b2	Ets1	Prtn3	Etv3	Cxcl13	Tcf4
Fabp5	H2-Ab1	Serpinb1a	Hmgn2	Syngr2	Cfp	Dnajc7
Prdx1	H2-Eb1	Jun	Wfdc21	Map4k4	Fam46a	Bst2
Clec4n	Id2	Tmem71	Lcn2	Tmem176a	Emilin2	Irf8
Spp1	H2-Aa	Ccl3	Hmgb2	AW112010	Pycard	Ly6c2
Retnla	Tmsb10	Rgs1	H2afz	Fabp5	Tagln2	Plac8
Lyz1	Cd74	Ccl9	S100a8	Epsti1	Slpi	Xist

Supplementary Figures

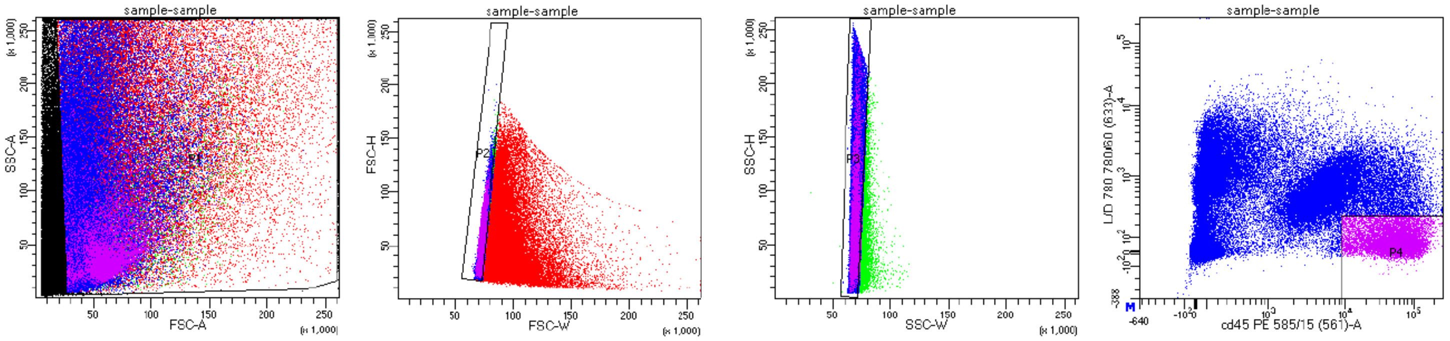

Tube: sample

Population	#Events	%Parent	%Total
All Events	500,000	####	100.0
P1	158,223	31.6	31.6
P2	89,526	56.6	17.9
P3	84,499	94.4	16.9
P4	10,076	11.9	2.0

Supplementary Figure 1

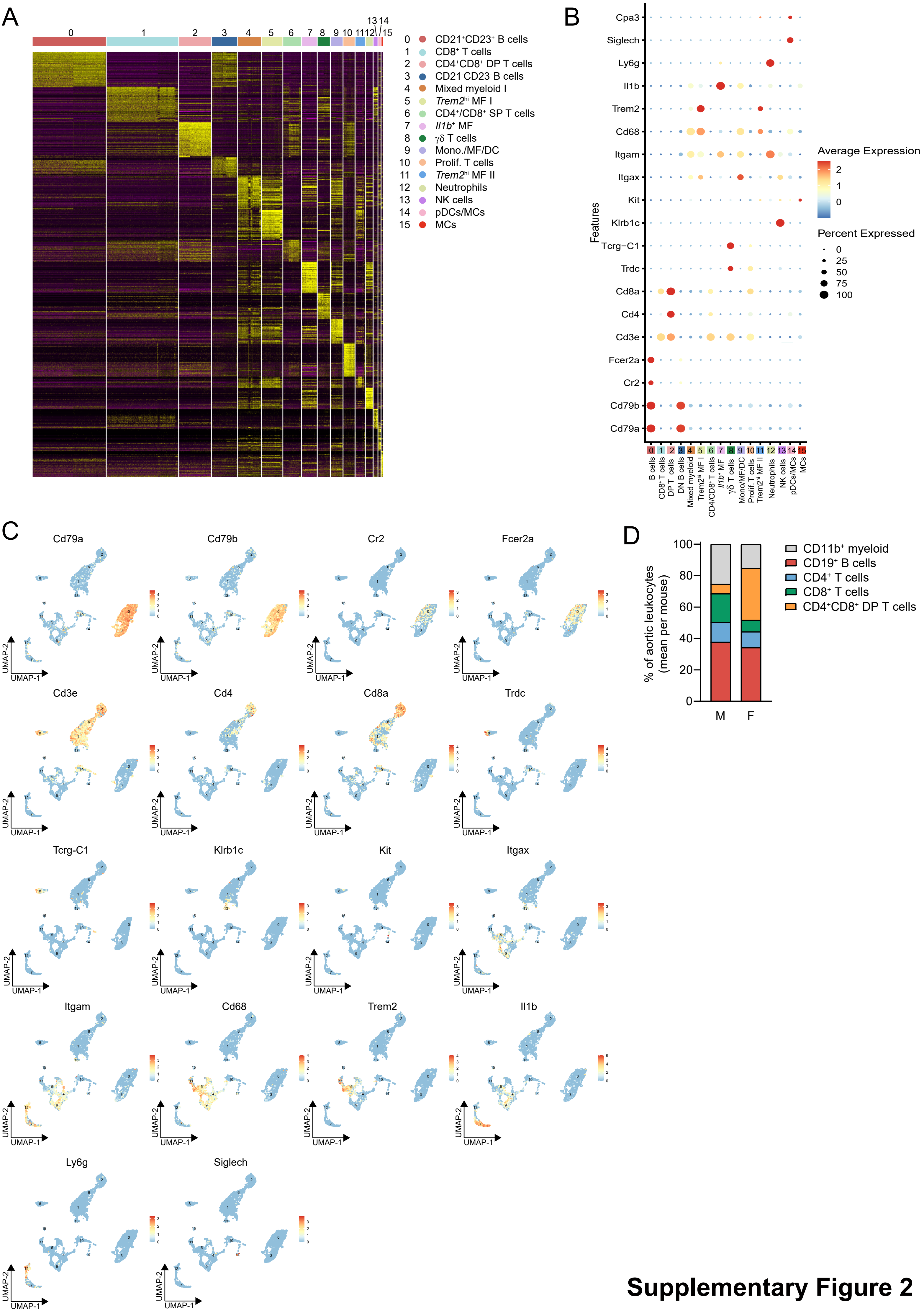

Supplementary Figure 2

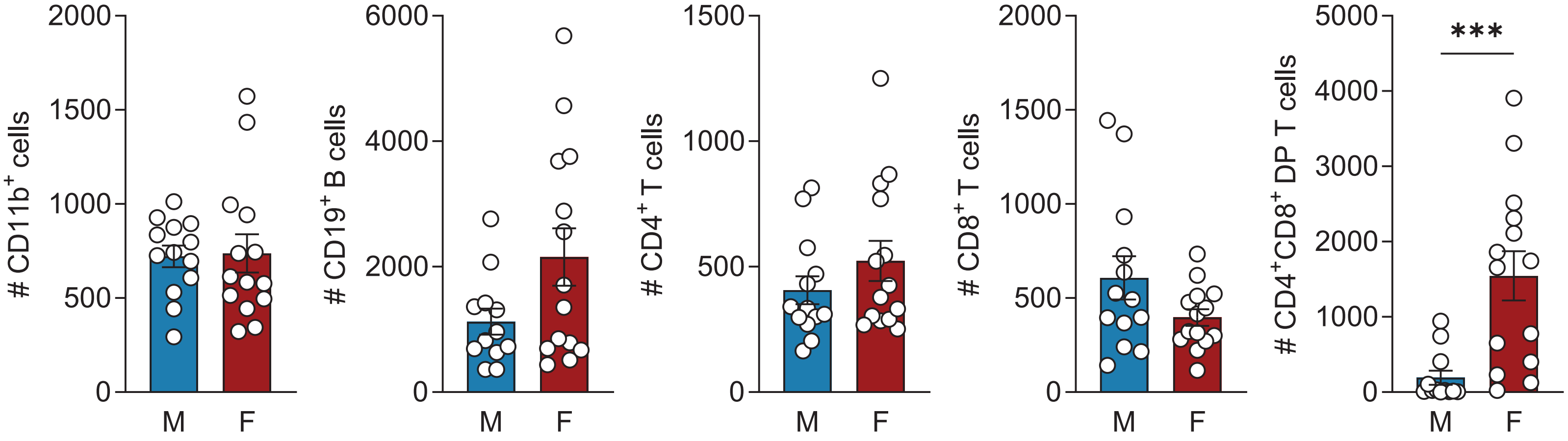

Supplementary Figure 3

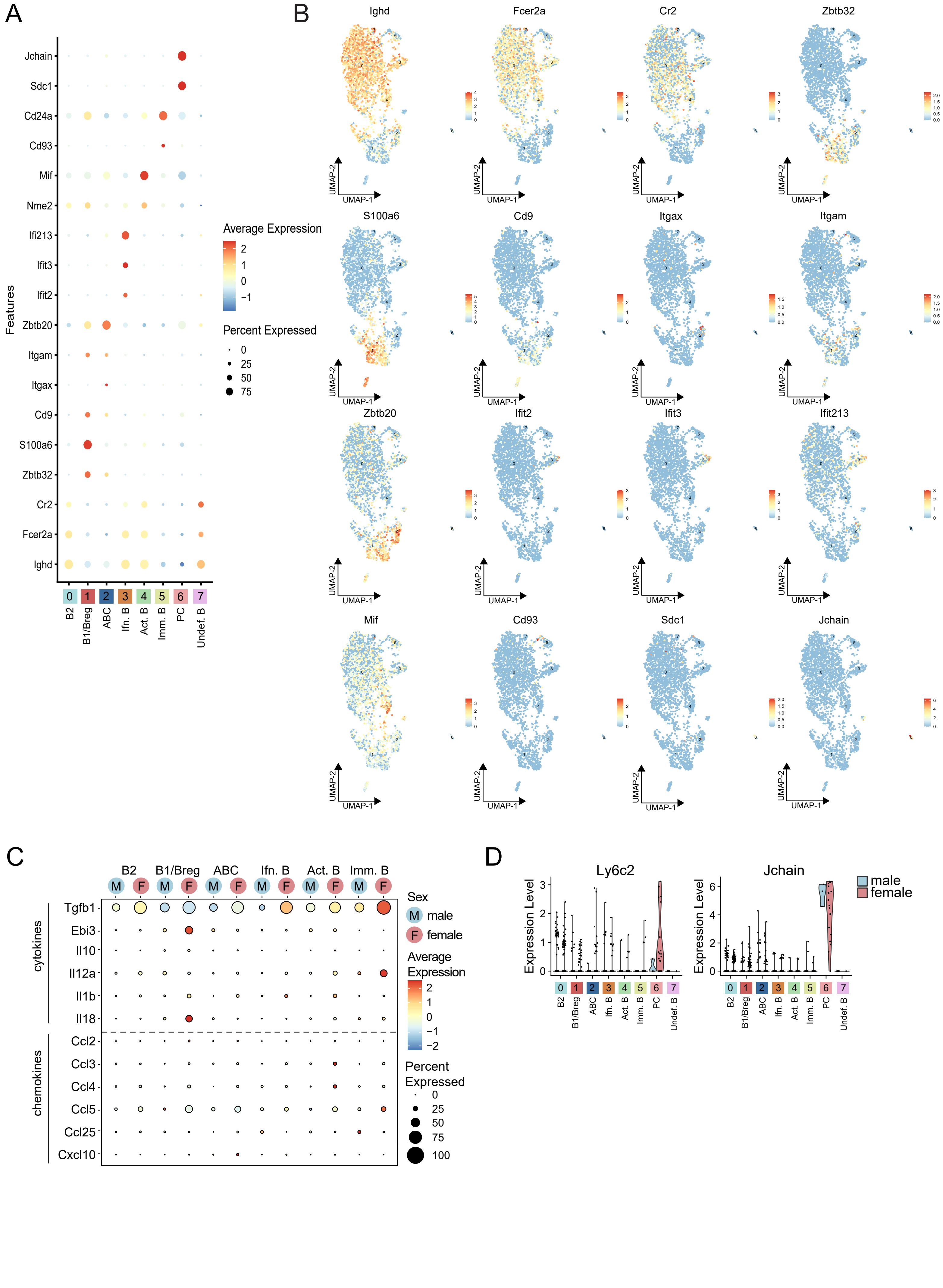

Supplementary Figure 4

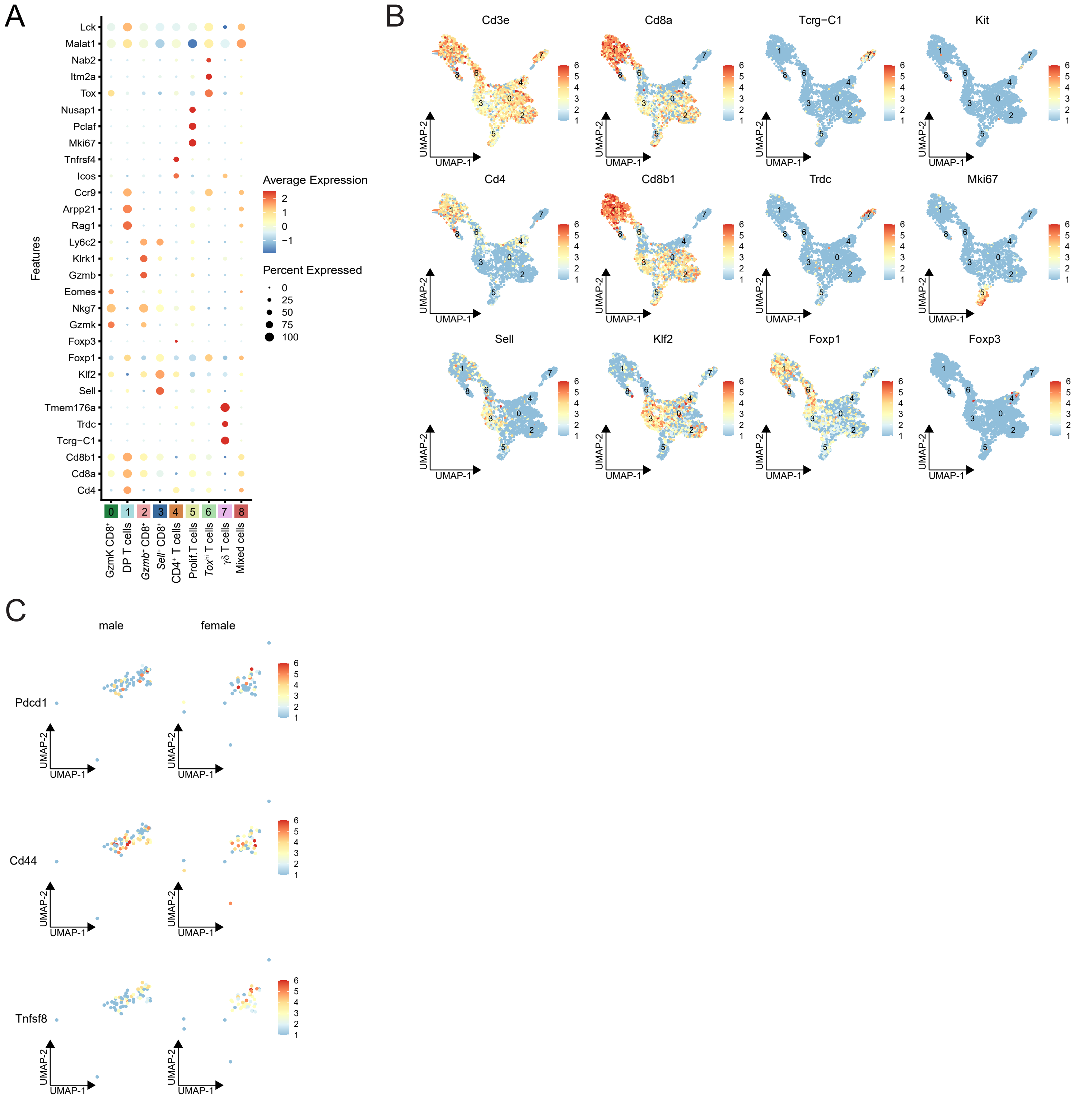

Supplementary Figure 5

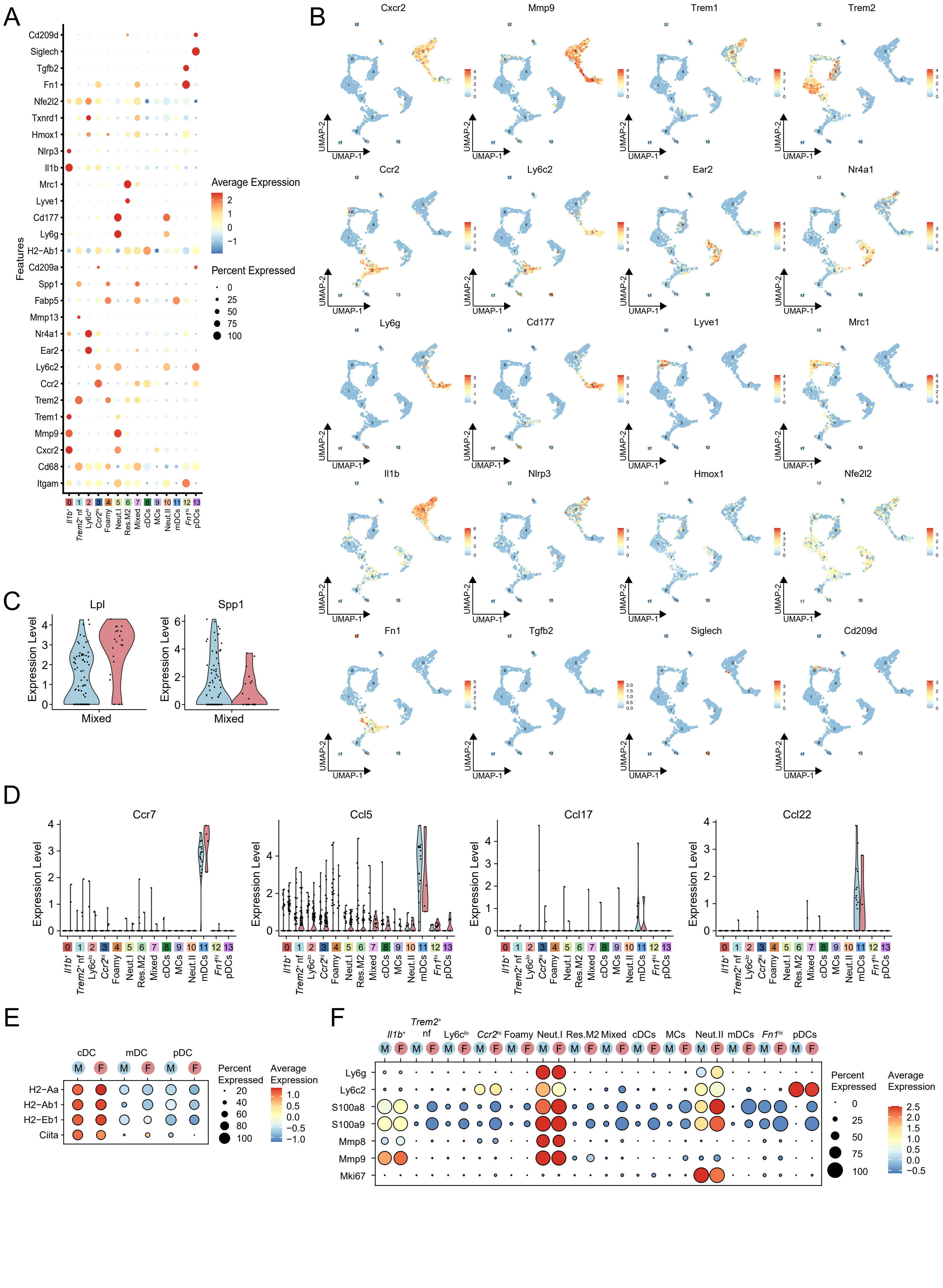

Supplementary Figure 6
